# Burn Mortality in an Appalachian Referral Center: An Examination of Mortality Prediction Scores in a 13-Year Retrospective Study

**DOI:** 10.7759/cureus.62912

**Published:** 2024-06-22

**Authors:** Armein Rahimpour, Nathan Fox, Grant Kahley, Paul Bown, David A Denning, Peter Ray, Rahman Barry

**Affiliations:** 1 General Surgery, Marshall University Joan C. Edwards School of Medicine, Huntington, USA; 2 Plastic Surgery, King’s Daughters Medical Center, Ashland, USA; 3 Plastic and Reconstructive Surgery, Marshall University Joan C. Edwards School of Medicine, Huntington, USA

**Keywords:** burn units, mortality risk factors in burn, modified baux score, rural appalachia, burn injury

## Abstract

Introduction

Burn injuries have profound implications, prompting the use of various mortality scoring systems. This study aimed to evaluate their effectiveness within our Appalachian burn referral center, which serves as the sole burn center in the state of West Virginia. Given this unique status, understanding the efficacy of mortality scoring systems within our center is crucial for resource allocation and optimizing patient outcomes in our region.

Methods

A retrospective analysis of patients admitted to Cabell Huntington Hospital Burn Intensive Care Unit (BICU) from January 2010 to June 2023 was conducted, assessing Baux (B), revised Baux (rB), Belgian Outcome in Burn Injury (BOBI), and Abbreviated Burn Severity Index (ABSI) scores. Logistic regression and receiver operating characteristic analysis were employed to examine survival status and determine optimal cut points.

Results

Among 1,104 patients, 57 died (5% mortality rate). Deceased patients had significantly higher B/rB/BOBI scores (mean: 98/98/92) than survivors (45/46/4.19) (p < 0.001), with ABSI showing no significance (p = 0.079). Each one-point increase in B/rB/BOBI scores correlated with a 1.09/1.09/2.34 times higher mortality risk (p < 0.001). The AUC for B score in predicting mortality was 0.926 (95% CI: 0.890, 0.962), with sensitivity and specificity values of 0.789 and 0.92, respectively, and an optimal cutoff point of 79. The AUC for the rB score was 0.927 (95% CI: 0.892, 0.962), with sensitivity and specificity values of 0.789 and 0.926, respectively, and an optimal cutoff point of 80. The AUC for the BOBI score was 0.901 (95% CI: 0.865, 0.937), with sensitivity and specificity values of 0.895 and 0.775, respectively, and an optimal cutoff point of 2. For patients with B scores above 79, their odds of mortality were 42.6 times higher than those with B scores of 79 or lower (95% CI: 22.6, 85.6, p < 0.001). Similarly, for patients with rB scores exceeding 80, their odds of mortality were 42.9 times higher than those with rB scores of 80 or lower (95% CI: 22.9, 84.8, p < 0.001). Finally, for patients with BOBI scores greater than 2, their odds of mortality were 17.8 times higher than those with BOBI scores of 2 or lower (95% CI: 9.88, 33.4, p < 0.001).

Conclusion

Our study underscores the vital role of mortality scoring systems in guiding clinical decision-making and resource allocation for burn patients, particularly within the Appalachian region served by the Cabell Huntington Hospital BICU. By leveraging tools such as the Baux, revised Baux, and BOBI scores, healthcare providers can identify high-risk patients early in their treatment course, facilitating personalized interventions and improving overall patient outcomes. Moreover, our findings highlight the significance of age and total body surface area burned as key determinants of mortality risk, emphasizing the need for tailored approaches to care for elderly patients and those with extensive burns. Continued research and refinement of mortality scoring systems are essential to further enhance their effectiveness and ensure optimal patient care in the challenging field of burn management.

## Introduction

Each year, an estimated 180,000 lives are lost to the devastating and challenging-to-treat injuries caused by burns [1]. Even if the initial burn injury is not fatal, it often leads to high morbidity rates, yet it is a largely preventable tragedy [1,2]. The financial burden is also substantial, with each burn patient costing the healthcare system approximately US$88,218 on average [1,2]. Despite advancements in technology, the prognosis of a severe burn injury remains poor [3]. Consequently, there is a significant focus on research and finding ways to alleviate this burden.

Various mortality prediction tools have been developed in efforts to enhance the treatment and management of burns more effectively. Research indicates that advanced age and increased burned surface area are correlated with higher mortality rates [4,5]. One of these predictive tools, the Baux score, leverages these factors for mortality prediction. It is used to predict the probability of survival in burn injuries. Studies regarding the efficacy and utility of the Baux score have been done to try and pinpoint its precision and accuracy in environments ranging from developing countries to pediatric populations, patients with inhalation injuries, and many more [5].

Acknowledging the considerable influence of inhalational injuries on burn mortality, the revised Baux score integrates an additional 17 points if an inhalational injury is identified [5]. Another mortality predictor score employed is the Abbreviated Burn Severity Index (ABSI) score. In addition to the factors considered in the revised Baux score, the ABSI score also accounts for gender and the presence of full-thickness burns [4,6]. Lastly, the Belgian Outcome for Burn Injury (BOBI) score for burn mortality prediction, like the revised Baux score, looks at age, total body surface area (TBSA) burned, and inhalational injury [6].

The Cabell Huntington Burn Intensive Care Unit (BICU) stands as a vital lifeline for the entire state of West Virginia, serving as its sole dedicated facility for burn care. However, its significance transcends mere geography; the many unique aspects of the patient population are crucial to understanding so they may better serve their patients’ needs. This population is disproportionately affected by cardiovascular disease and cancer, among other comorbidities, and these patients are more likely to make deleterious health choices [7]. In general, patients from rural regions have decreased access to quality care, whether that be due to a lack of providers in the area, increased travel, or financial burden [8].

This study aimed to assess the usefulness and accuracy of different mortality scores, specifically in the Appalachian area, as well as identify what factors increase the mortality risk in burn patients in our area. The use of these burn injury severity ranking tools and further knowledge of the factors that influence burn recovery outcomes can help Appalachian healthcare workers best allocate treatments and resources to patients with severe burns. This information will be best suited to allow for early detection and prevention of factors that would have traditionally led to poor outcomes.

## Materials and methods

The study received approval from the Marshall University Institutional Review Board (approval number 2063568-1). Patient records were retrospectively reviewed from our registry at Cabell Huntington Hospital Burn Care Intensive Unit, West Virginia’s sole BICU. Cabell Huntington Hospital is an academic teaching hospital, regional referral center, and American College of Surgeons-verified Level-2 Trauma Center in Huntington, WV.

The analyzed medical files belonged to patients who presented between January 1, 2010, and June 1, 2023. Data were obtained by contacting the information technology (IT) department. The request included any patient who presented to the hospital with burns. The inclusion criteria were burn patients presenting to the hospital. Additionally, we requested age, gender, Injury Severity Score (ISS), and hospital length of stay from the IT team. The initial sample consisted of more than 1,300 patients. All collected data was centralized using Microsoft Excel software (Microsoft Corporation, Redmond, United States). Medical records for those patients were reviewed, and data on the percentage of TBSA, presence of inhalation injury, and degree of burn were collected. The inhalational injury was diagnosed with the presence of carbonaceous material or soot in the oropharynx with difficulty in oxygenation. The initial sample of patients obtained from IT services included all patients with a diagnosis of burn. However, patients with a misdiagnosis of burns upon chart review were excluded. These patients included those with Stevens-Johnson syndrome, road rash, frostbite, or no documentation of burns noted in the chart review. After the chart was reviewed, the final sample consisted of 1,104 patients.

To collect and calculate the four different mortality scores for burn injuries, we utilized established prediction models and scoring systems, as mentioned in the introduction section. These systems were developed based on various risk factors associated with burn injuries, such as TBSA, age of the patient, presence of an inhalation injury, and other demographic variables.

The Baux score was calculated using two factors, the first being the TBSA plus the age of the patient [5]. The TBSA was calculated using the Wallace rule of nines [9]. To calculate the revised Baux score, if the inhalational injury was positive, 17 was added to the Baux score [5]. The mortality score for the Baux score and the revised Baux score are equal. To calculate the ABSI, five different factors were used: sex, age, presence of inhalation injury, presence of a full-thickness burn, and TBSA (Table 1) [10]. Depending on the score, the probability of survival was calculated (Table 2) [10].

**Table 1:** ABSI score ABSI, Abbreviated Burn Severity Index

Parameter	Finding	Points
Sex	Female	1
	Male	0
Age (years)	0–20	1
	21–40	2
	41–60	3
	61–80	4
	81–100	5
Inhalation injury	Yes	1
	No	0
Presence of a full-thickness burn	Yes	1
	No	0

**Table 2:** ABSI score and mortality prediction ABSI, Abbreviated Burn Severity Index

ABSI	Probability of survival (%)
2-3	≥99
4-5	98
6-7	89-90
8-9	50-70
10-11	20-40
≥12	≤10

Finally, to calculate the BOBI score, age, TBSA, and inhalational injury were used (Table 3) [11]. Depending on the score, mortality was predicted (Table 4) [11].

**Table 3:** BOBI score BOBI, Belgian Outcome in Burn Injury; TBSA, total body surface area

Parameter	Finding	Points
Age (years)	<50	0
	50-64	1
	65-79	2
	>80	3
Inhalation injury	Yes	3
	No	0
TBSA burn (%)	<20	0
	20-39	1
	40-59	2
	60-79	3
	80-100	4

**Table 4:** BOBI score and mortality prediction BOBI, Belgian Outcome in Burn Injury

BOBI	Mortality (%)
0	0.1
1	1.5
2	5
3	10
4	20
5	30
6	50
7	75
8	85
9	95
10	100

Descriptive statistics were used to summarize the sample characteristics. Continuous variables were expressed as means ± SDs, while categorical variables were presented as numbers (N) and percentages (%). The chi-square test was utilized to determine significant differences between the two groups based on survival status for each categorical variable. Fisher’s exact test was used instead when the expected count was less than 5. For numeric variables, a Student’s t-test was applied. Logistic regression models were applied to assess the association between each predictor and survival status. Furthermore, receiver operating characteristic (ROC) analysis was utilized, specifically based on the Youden Index, to identify the optimal cut point. This optimal cut point was subsequently utilized in conjunction with the Baux score, modified Baux, Belgian Outcome in Burn Injury (BOBI) score, and logistic regression models to investigate and assess their association with the outcome of survival status. All statistical analyses were conducted using SAS (SAS 9.4, SAS Institute Inc., Cary, United States). Statistical significance was defined using a two-sided test with a p-value <0.05.

## Results

Table 5 provides an overview of the demographic and clinical characteristics of patients admitted to the Cabell Huntington Hospital BICU, stratified by their survival status. The gender distribution in the cohort, consisting of 1,104 individuals, revealed that 329 (30%) were female, with the remaining 775 (70%) being male. These gender proportions remained consistent within the subsets of survivors (N = 1,047) and deceased patients (N = 57), with no statistically significant disparities observed between the two groups. The average age of the entire cohort was 40 years. Among the survivors, the mean age was 39 years, while among those who did not survive, the average age notably increased to 66 years (p < 0.001). As for the deceased patients, the Baux score was significantly higher when compared to the surviving patients. Deceased patients had a mean Baux score of 98 with an SD of 27, whereas survivors exhibited a mean of 46 with an SD of 25 (p < 0.001). Similarly, the revised Baux score was notably higher for deceased patients, with a mean of 98 with SD 27, while surviving patients had a mean of 46 with SD 25 (p < 0.001). Furthermore, the BOBI scores demonstrated significant differences. Survivors had a mean BOBI score of 0.92 with SD 1.37, whereas deceased patients exhibited a substantially higher mean BOBI score of 4.19 with SD 2.13 (p < 0.001). In contrast, the ABSI did not exhibit a statistically significant difference between surviving and deceased patients (p = 0.079). Survivors had a mean ABSI of 4.62 with SD 1.92, while deceased patients had a mean ABSI of 4.09 with SD 1.30, suggesting a relatively similar severity index for both groups in this context.

**Table 5:** Sample characteristics by survival status Data are presented as N (%), excluding SD. ABSI, Abbreviated Burn Severity Index; BOBI, Belgian Outcome in Burn Injury

Variables	Overall (N = 1,104)	Alive (N = 1,047)	Dead (N = 57)	p-value
Gender				0.55
Female	329 (30)	310 (30)	19 (33)	
Male	775 (70)	737 (70)	38 (67)	
Age				<0.001
Mean ± SD	40 ± 23	39 ± 23	66 ± 14	
Baux				<0.001
Mean ± SD	49 ± 27	46 ± 25	98 ± 27	
Revised Baux				<0.001
Mean ± SD	49 ± 27	46 ± 25	98 ± 27	
ABSI				0.079
Mean ± SD	4.59 ± 1.90	4.62 ± 1.92	4.09 ± 1.30	
BOBI				<0.001
Mean ± SD	1.09 ± 1.59	0.92 ± 1.37	4.19 ± 2.13	

Table 6 presents the outcomes of logistic regression models, examining the relationship between various predictor variables and survival status. Both the Baux score and revised Baux score were significantly associated with survival status, with each one-point increase in these scores linked to a 1.09 times higher risk of mortality (p < 0.001). The BOBI score is also a significant predictor, with a one-unit increase associated with a 2.34 times higher risk of death (95% CI: 2.02, 2.77, p < 0.001).

**Table 6:** Association between predictors and survival status by using the logistic regression models BOBI, Belgian Outcome in Burn Injury

Variables	OR	95% CI	p-value
Baux	1.09	1.07, 1.11	<0.001
Revised Baux	1.09	1.07, 1.11	<0.001
BOBI	2.34	2.02, 2.77	<0.001

**Figure 1:**
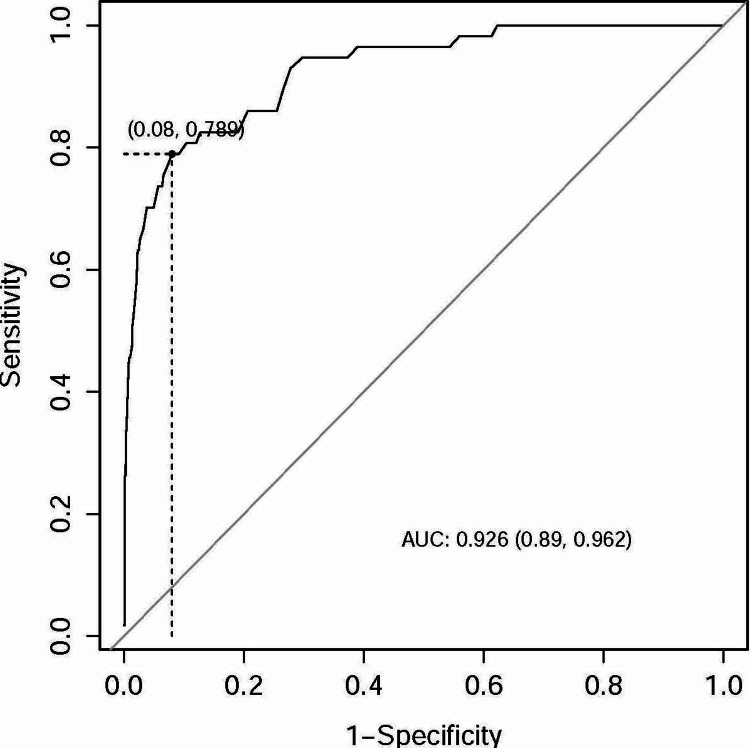
ROC curve for the Baux prediction model ROC, receiver operating characteristic

**Figure 2:**
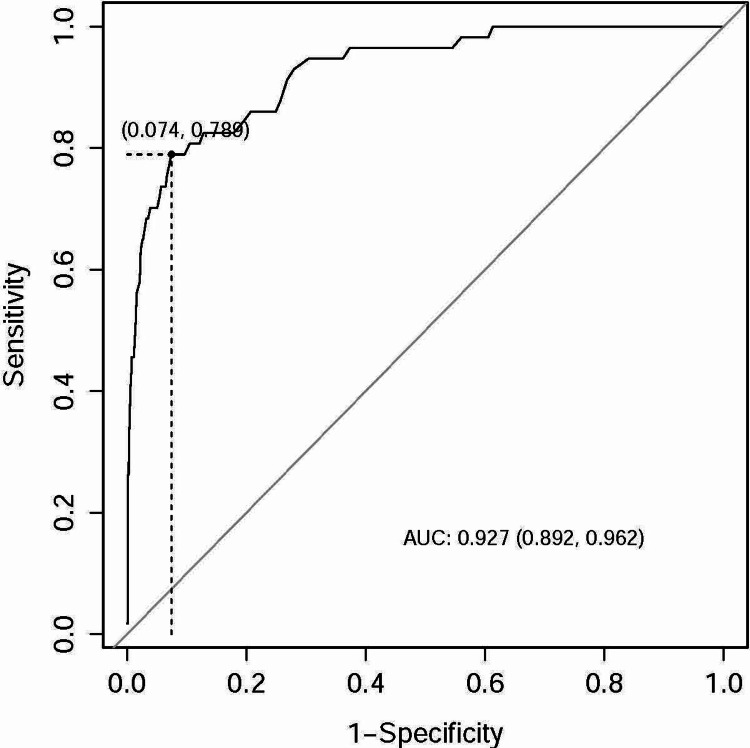
ROC curve for the revised Baux prediction model ROC, receiver operating characteristic

**Figure 3:**
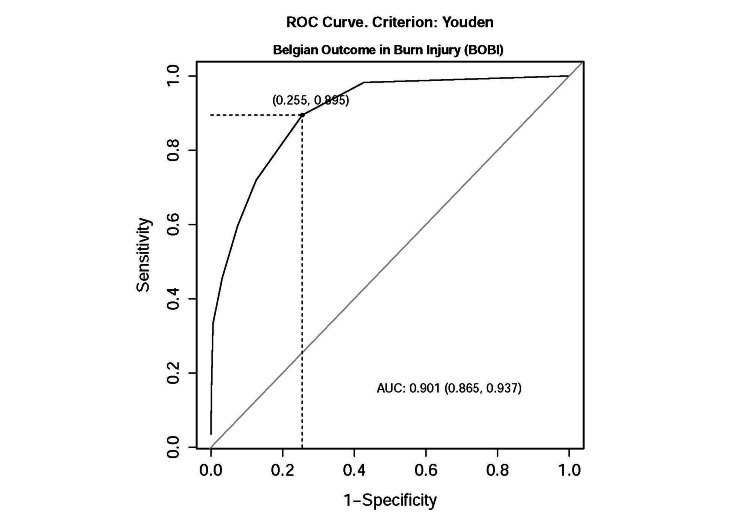
ROC curve for the BOBI prediction model BOBI, Belgian Outcome in Burn Injury; ROC, receiver operating characteristic

Furthermore, ROC analysis was conducted to ascertain optimal cutoff points using Youden's criteria. The area under the curve (AUC) for the Baux score in predicting mortality was 0.926 (95% CI: 0.890, 0.962), with sensitivity and specificity values of 0.789 and 0.92, respectively, and an optimal cutoff point of 79 (Figure 1). The ROC analysis for the revised Baux score yielded similar results, with an AUC of 0.927 (95% CI: 0.892, 0.962), sensitivity and specificity values of 0.789 and 0.926, respectively, and an optimal cutoff point of 80 (Figure 2). The BOBI score, however, had a lower AUC compared to the Baux score and revised Baux score, with an AUC of 0.901 (95% CI: 0.865, 0.937), sensitivity and specificity values of 0.895 and 0.775, respectively, and an optimal cutoff point of 2 (Figure 3).

In Table 7, the association between predictors and survival status with optimal cutoff points is shown. For patients with Baux scores above 79, their odds of mortality were 42.6 times higher than those with Baux scores of 79 or lower (95% CI: 22.6, 85.6, p < 0.001). Similarly, for patients with revised Baux scores exceeding 80, their odds of mortality were 42.9 times higher than those with revised Baux scores of 80 or lower (95% CI: 22.9, 84.8, p < 0.001). Finally, for patients with BOBI scores greater than 2, their odds of mortality were 17.8 times higher than those with BOBI scores of 2 or lower (95% CI: 9.88, 33.4, p < 0.001).

**Table 7:** Association between predictors and survival status by using the logistic regression models and optimal cutoff points BOBI, Belgian Outcome in Burn Injury

Variables	OR	95% CI	p-value
Baux score ≤79	1	Ref	Ref
Baux score >79	42.6	22.6, 85.6	<0.001
Revised Baux score ≤80	1	Ref	Ref
Revised Baux score >80	42.9	22.9, 84.8	<0.001
BOBI ≤2	1	Ref	Ref
BOBI >2	17.8	9.88, 33.4	<0.001

## Discussion

Interpretation of the findings

This study underscores the propensity for males to experience burn, although it did not reveal significant gender-based disparities in survival rates. While the ABSI failed to serve as a significant predictor of mortality within our population, this might be attributed to its categorical inclusion of gender. As observed in other research [1,4], burns tend to be more prevalent among males, as corroborated by our findings. However, contrary to expectations based on previous studies, our research did not identify higher mortality rates among females [1,4]. This deviation could be elucidated by the unique demographic served by our institution, the Cabell Huntington BICU, being the sole BICU in the state of West Virginia. Our patient population reflects the geographical and socioeconomic realities outlined by WHO, wherein individuals in lower and middle-income brackets face heightened risks of burns due to occupational hazards, alcohol misuse, and exposure to combustible substances [4].

The study’s findings provide compelling evidence regarding the effectiveness of mortality scoring systems in predicting outcomes for burn patients, particularly within the unique context of the Appalachian region, specifically within the Cabell Huntington Hospital BICU. The identified correlations between elevated Baux, revised Baux, and BOBI scores with heightened mortality risks emphasize the clinical utility of these scoring systems in informing treatment strategies and resource allocation, all of which factor in age and TBSA.

Our investigation highlighted age as an independent determinant of survival, with the average age of non-surviving patients being 66 years, corroborating existing research [1,2,4]. An average age of >60 years was seen to exhibit the highest mortality rates in another study [4]. This phenomenon can be partly attributed to age-related variations in the inflammatory response to dermal injuries [12], underlying comorbidities [13], and diminished physiological reserves among the elderly [4]. Literature suggests tailored approaches for elderly burn patients, emphasizing non-aggressive resuscitation, consideration of physiological age, and a methodical surgical approach to minimize anesthesia-related complications and blood loss, followed by comprehensive rehabilitation programs comprising vigorous physical and occupational therapy [14]. Anecdotal evidence from our practice suggests that elderly burn victims who receive consistent familial support tend to fare better. Moreover, a higher TBSA burn correlated with increased mortality risk, consistent with established literature [1,4,15], with 100% mortality observed in patients with 60% TBSA burns [15].

Clinical implications and outcomes

The study’s findings have important implications for clinical practice within the Cabell Huntington Hospital BICU and similar healthcare settings. By leveraging mortality scoring systems like the Baux, revised Baux, and BOBI scores, healthcare providers can identify high-risk patients early, allowing for timely interventions and resource allocation. Our investigation, reflected in the ROC analysis, aligns closely with previous research, showcasing the robust predictive capacity of these scoring systems, albeit with some limitations in extreme age groups [16]. International validation from studies in Japan and Malaysia further bolsters the applicability and reliability of these scores across diverse patient populations [17,18].

The determination of optimal cutoff points through ROC analysis enhances the clinical utility of these scoring systems, enabling healthcare providers to establish thresholds for identifying patients at significantly elevated risk of mortality. This risk stratification approach facilitates personalized treatment planning and may contribute to improved patient outcomes. This study holds particular relevance for healthcare practitioners in West Virginia, given the unique status of the BICU in the state. The establishment of new optimal cutoff points equips rural clinicians with enhanced tools for efficiently allocating resources, benefiting patients within the BICU, and aiding in the seamless transfer of patients between healthcare facilities.

One key clinical outcome highlighted by this study is the significant association between higher mortality scores and an increased risk of death among burn patients. Patients with elevated Baux, revised Baux, and BOBI scores exhibited substantially higher odds of mortality, emphasizing the predictive power of these scoring systems. Moreover, the findings underscore the importance of ongoing evaluation and refinement of mortality scoring systems in clinical practice. Continuous monitoring of patient outcomes and recalibration of scoring systems are essential to ensure accuracy and effectiveness in predicting mortality risk among burn patients.

Efficient resource allocation is critical in burn care, particularly in resource-constrained settings like the Appalachian region served by Cabell Huntington Hospital. The study’s findings enable healthcare providers to prioritize interventions for high-risk patients based on their scoring system-derived risk profiles, thereby optimizing patient care and resource utilization within the BICU.

Limitations and future research

While the findings of this study are promising, several limitations must be acknowledged. The retrospective design introduces potential biases and may limit the generalizability of the findings to other healthcare settings or patient populations. Collaborative multicenter studies involving diverse patient populations are needed to validate the findings and enhance their external validity. Furthermore, while the study evaluated multiple mortality scoring systems, additional research is warranted to explore the integration of novel predictive factors or biomarkers into existing scoring systems. Prospective validation studies in real-time clinical settings are needed to confirm the efficacy of these scoring systems and refine optimal cutoff points based on real-world data. Longitudinal studies tracking patient outcomes beyond the acute phase of burn injury are also needed to understand the long-term implications of mortality scoring systems and inform comprehensive patient care strategies.

## Conclusions

Our study underscores the vital role of mortality scoring systems in guiding clinical decision-making and resource allocation for burn patients, particularly within the Appalachian region served by the Cabell Huntington Hospital BICU. By leveraging tools such as the Baux, revised Baux, and BOBI scores, healthcare providers can identify high-risk patients early in their treatment course, facilitating personalized interventions and improving overall patient outcomes. Moreover, our findings highlight the significance of age and TBSA burns as key determinants of mortality risk, emphasizing the need for tailored approaches to care for elderly patients and those with extensive burns. Continued research and refinement of mortality scoring systems are essential to further enhance their effectiveness and ensure optimal patient care in the challenging field of burn management.
